# Supplementary material for: Upregulation of Claudin-7 Expression by Angiotensin II in Colonic Epithelial Cells of Mice Fed with NaCl-Depleted Diets
Source: Int J Mol Sci. 2020 Feb 20;21(4):1442. doi: 10.3390/ijms21041442 (PMC7073026; doi:10.3390/ijms21041442)
Supplement: Supplementary file 1 [file ijms-21-01442-s001.pdf]

-1491	-1481	-1471	-1461	-1451	-1441
ACTAGAAAGA	GTTCAGGGGG	GCAGTGATGG	ACACACTGGG	CTACTGGGCT	CCAAGGGTCT
-1431	-1421	-1411	-1401	-1391	-1381
GGAGGGAGCA	ACCTTAGTCT	GGAAGTGTGT	GTGTGTGTGT	GTGTGTGTGT	GTGTGTGTGT
-1371	-1361	-1351	-1341	-1331	-1321
GTGTGTGTGT	TGGGGGGGGT	GAATAAAAAT	GCAGAAGGCA	GGAAAAGGGG	AAAGATAAAT
-1311	-1301	-1291	-1281	-1271	-1261
GAGAACCTTA	GAAGCCAAGC	TGGGAGGAAG	GATGGTCAAA	GTTCTGGGAA	AGCTGGAAGG
-1251	-1241	-1231	-1221	-1211	-1201
AGTGGAGGCT	GAGCTAGGCT	TTCTATTTTT	TTTTTTAAGA	TTTTATTATT	ATATTTAAAT
-1191	-1181	-1171	-1161	-1151	-1141
ACACTGTAGC	TGTCTTCAGA	CACACCAGAA	GAGGGTGTCA	AGATCTCATT	ATGGATGGTT
-1131	-1121	-1111	-1101	-1091	-1081
GTGAGCCACC	ATGTGTTTAC	TGGGACTTGA	ACTCAGGACC	TTTGGAAGAG	CAGTCAGTGC
-1071	-1061	-1051	-1041	-1031	-1021
TCTTAACCCC	TGAGCCATCT	CCCCAGCCTT	AAGCTAGACT	TTCTAAGGGA	TGAATTAAGA
-1011	-1001	-991	-981	-971	-961
TGCTAGAGTG	GGTCGGAGGG	CACCGAAAGG	GCACACAAAC	AGGTCCCCGG	AAGCTCAGAA
-951	-941	-931	-921	-911	-901
TGAAGGAAAG	AGAAAGTGTG	TGGATGGAGA	AGGAAGTGAG	GAAAATGAAG	GTAAACCAGA
-891	-881	-871	-861	-851	-841
AATGACAACC	TGGGGAGGGG	AATGTTGGGC	GTGTCGTGTC	AGACTCTCTG	ACCTAGGTCT
-831	-821	-811	-801	-791	-781
TACCCTTCTC	CCCACCCTCG	GAAAACTCCT	TCGGGTAGAA	GGAGCCTCTA	TGTTGGAGGA
-771	-761	-751	-741	-731	-721
GACACTGACA	GGCTCAGGTG	CTAAGTCCTG	TACCTACCTG	GTCCTGGGAC	CAGGCCTGTG
-711	-701	-691	-681	-671	-661
GGAGGACTGT	CGGAGAGCCG	TGTCTTGTGG	AGGGCTTGAG	CGGTGAAGCG	GTAGGTTGCT
-651	-641	-631	-621	-611	-601
AGACTGTCTG	AGAGTAGAAA	TTCCTAGAAG	GGTGCATGCA	GCACGGGTGA	TTTTACCGTA
-591	-581	-571	-561	-551	-541
ATGGGTTAGG	GCCCCTGATG	ATGAGACAGG	CCTTGGTAGG	AGACTAAAGA	AAAAAAGAGC
-531	-521	-511	-501	-491	-481
TGAGAGTGGA	GACGAAAGTC	GGGCTGGACA	AGTGGCACGC	GCCGAAACCT	GCAGGGGAAG
-471	-461	-451	-441	-431	-421
GATGGCGAGG	AGCCTTTCCA	GGGGCGTCTG	AGGGTACTGG	CTCCGCAGCG	ACCCAGGCGC
-411	-401	-391	-381	-371	-361
ACCTGTATGG	AAAGGAGAGG	AAGGGGCCAC	TTGGAGTCTG	CAGGGCAGAC	TCCGACCCCA
-351	-341	-331	-321	-311	-301
GGGACTTCTG	AGAAGCCGTC	GCCTATTTCC	CATCAACGGT	TAGAGCCAGG	CAAATGAAGG
-291	-281	-271	-261	-251	-241
GGCGTGACCC	TGGAGCTCAG	GTTTCTTCCT	CTTCACCTGG	ACGAGGAGGG	GGTAGGGGCC
-231	-221	-211	-201	-191	-181
AAGACTTCCG	GTTCAGGTGA	GTGTCCCTTC	AGTGACGTCA	GGTCACTCGA	CTGCCCCTCT
-171	-161	-151	-141	-131	-121
GGTCCCGCCC	AAGTTGCAGC	GCTCCGGGTG	CCTGCGGGGG	CGCGTCCCCG	ACGTCCTGCA
-111	-101	-91	-81	-71	-61
TATATATACT	CAGGTGCGCC	GCACCTGCTC	GCCCGCACCT	GCCGCCGCAC	CGCCAGCTCC
-51	-41	-31	-21	-11	-1
CTGTGCCGCG	CACCGCAGCC	TGGGGCCCAA	GGGCCCGCAT	ACTTTCTGGG	GGCCACGCCC

Supplementary figure 1. Sequence of the promoter region of mouse CLDN7. Red boxes indicate presumable sites of κB action.
